# Supplementary material for: Biophysical properties of IgD determine thresholds for self-tolerance and selection into germinal centers
Source: bioRxiv. 2026 Jul 7:2026.07.02.735902. Preprint. [Version 1] doi: 10.64898/2026.07.02.735902 (PMC13370332; doi:10.64898/2026.07.02.735902)
Supplement: Supplement 1 [file NIHPP2026.07.02.735902v1-supplement-1.pdf]

Supplementary data fig. 1: Fab arm dynamics of IgM and IgD hinges measured by smFRET.

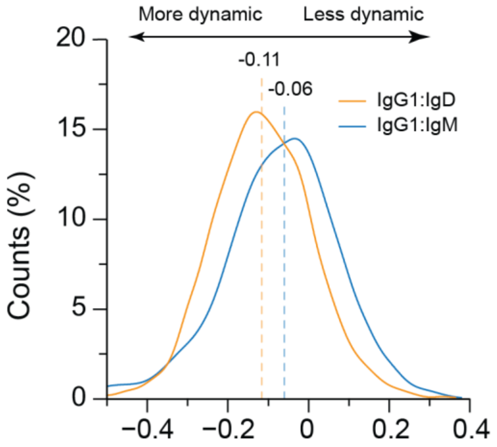

Histograms of Pearson's correlation of donor and acceptor intensities for IgG1:IgD (4332 molecules) and IgG1:IgM (4291 molecules) from data collected at 10 ms time resolution. Dotted lines denote the median.

Supplementary data fig. 2: Differential avidity effects of IgD and IgM in antigen capture.

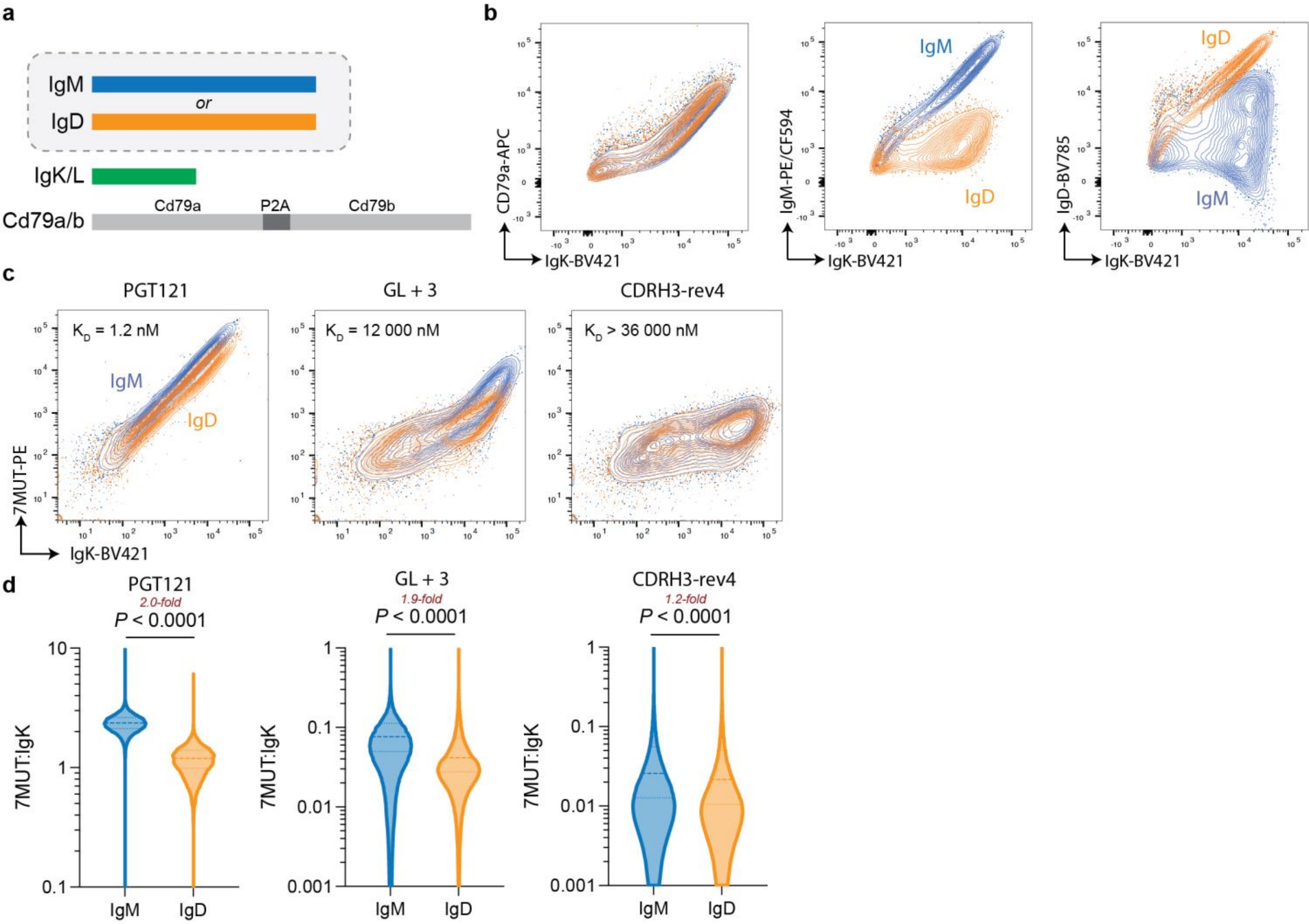

a, Depiction of the three-plasmid BCR transfection system comprised of plasmids encoding the murine light-chain, Cd79a/b, and either native membrane-bound IgM or IgD. The clone used in each panel are defined. HEK293 T cells were transiently transfected. **b,** Cells were transfected to express the gp120 (7MUT)-binding clone, PGT121, expressed as IgM or IgD BCR. Expression of the respective BCRs were confirmed by flow cytometry. **c,d,** BCR expression of PGT121 and their low-affinity partial inferred germline revertants, GL+3 and CDRH3-rev4 [33], was induced by transient transfection. **c,** Representative flow plots and **d,** violin plots show the relative dextramerized 7MUT 20-mer capture by cells with respect to isotype. Data were compared using a lognormal t-test.

Supplementary data fig. 3: Truncation of the IgD hinge rescues BCR antigen capture.

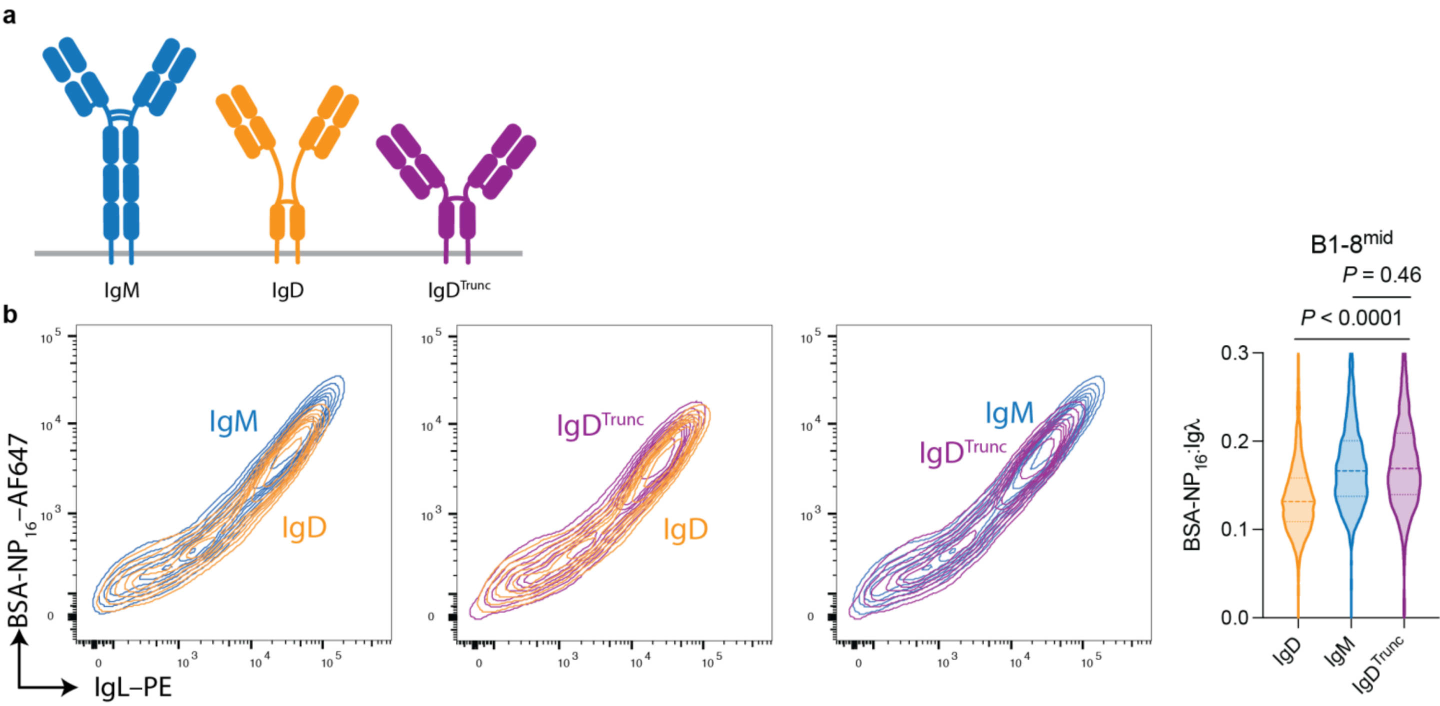

a, Graphical depiction of IgD^{Trunc} alongside native murine IgD and IgM BCR. IgD^{Trunc} was engineered to remove the 35 a.a. hinge of IgD (Δ94–128 a.a.; Uniprot: P01881). **b,** HEK293T cells were transiently transfected to express the NP-binding antibody B1-8^{mid} as a BCR (IgH, IgL and Cd79a/b). Antigen capture was quantified by flow cytometry using fluorescently-labelled BSA-NP$_{16}$ and normalizing to the surface BCR density. Representative flow plots show comparative antigen binding with respect to heavy chain, and violin plots show the ratio of antigen- and IgL-associated fluorescence. *P*-values were determined by Tukey's post-hoc comparison.

Supplementary data fig. 4: Differential binding of IgM and IgD against fixed antigen.

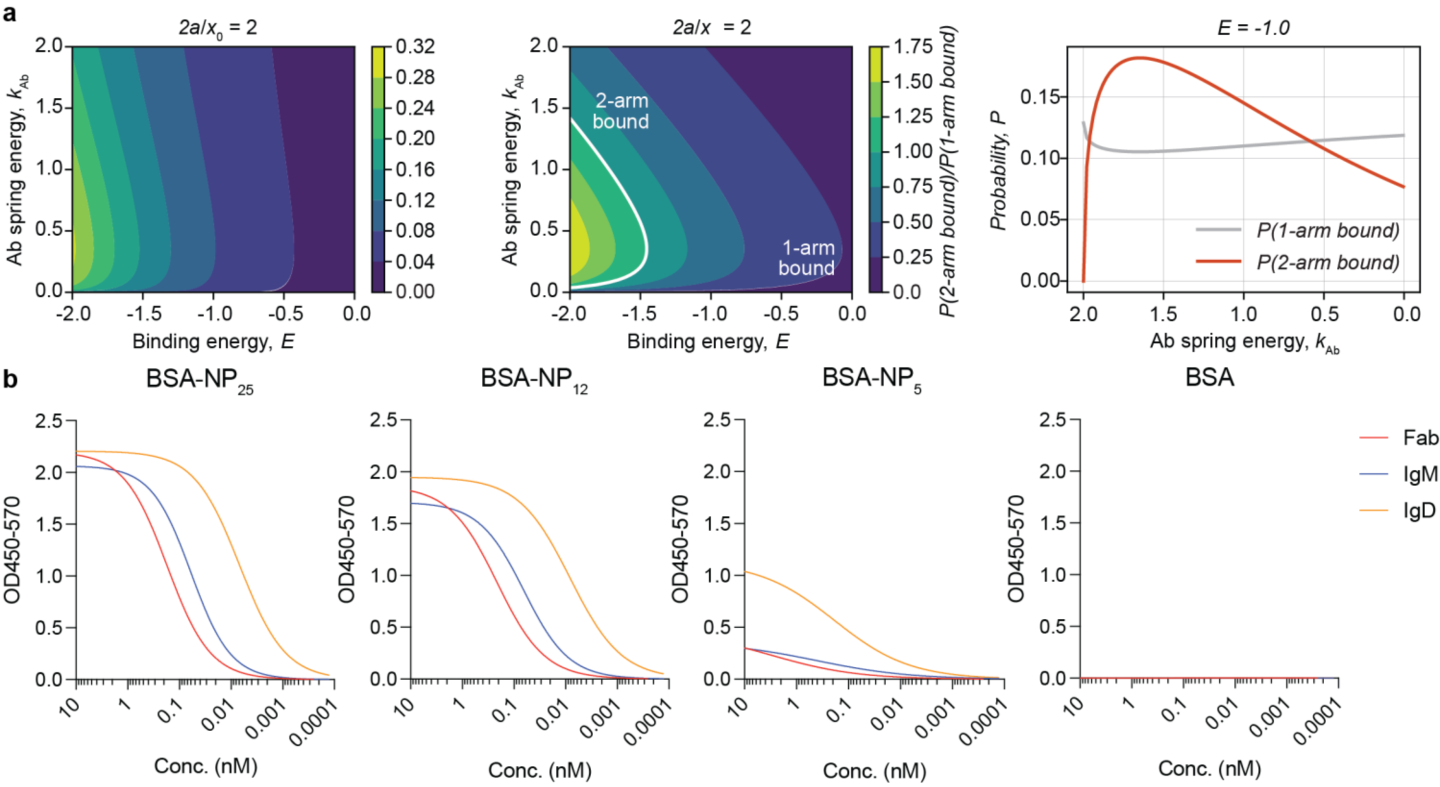

a, Plots show the probability of Ig binding to immobilized antigen that deviates from the preferred topology ($2a/x_0 \neq 1$), specifically where epitopes are more distant ($2a/x_0 > 1$). **b,** Plots show the ELISA traces of soluble B1-8^{hi} expressed as Fab, murine IgM and IgD binding to BSA-NP_{25}, or BSA-NP_{12}, or BSA-NP_5, or BSA. Traces reflect the averaged logistic regression function (n = 2).

Supplementary data fig. 5: Immune phenotyping of MD-, M- and D-only mice.

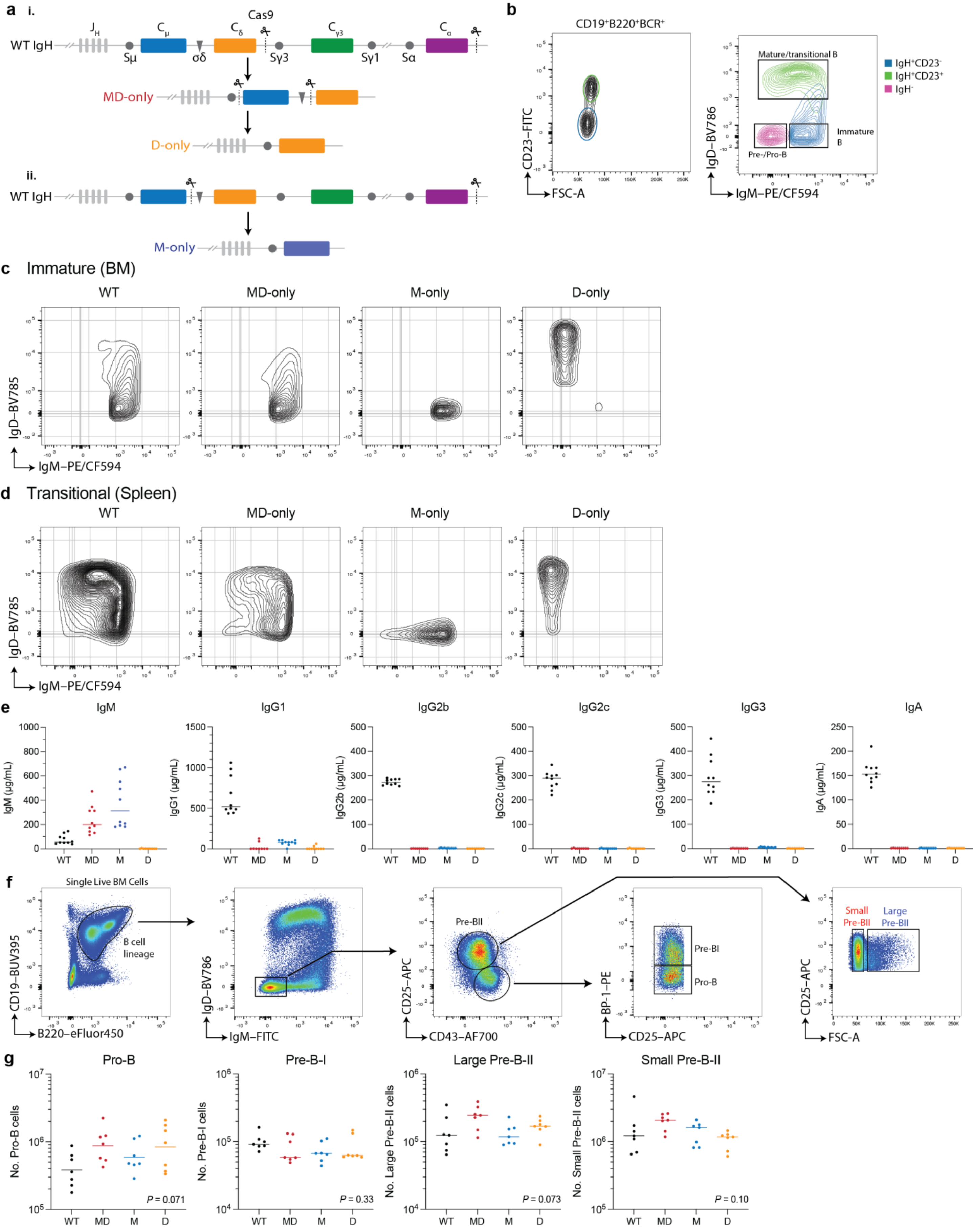

Supplementary data fig. 5: Immune phenotyping of MD- M- and D-only mice.

a, Graphical depiction of the *Igh* loci and Cas9 gene segment excision strategy to produce i) MD-only mice and subsequently D-only mice by a second round of targeting, and ii) M-only mice. See methods section. **b,** Representative flow cytometry plots of wild-type bone marrow B cells show that B220^{+}BCR^{+}CD23^{-} immature B cells (IgM$^{mid–hi}$IgD^{-}). Flow cytometry plots show the IgM and IgD expression profiles of **c,** bone marrow immature (BCR^{+}CD23^{-}) and **d,** splenic transitional (CD93^{+}) B cells from WT, MD-only, M-only and D-only mice. **e,** Plots show the serum concentration of Ig isotypes as determined by ELISA. **f,** Representative flow cytometry plots show the gating strategy used to identify early B cell precursors in the bone marrow. **g,** Dot plots show the number of B cell precursors per femur. Data were compared by one-way ANOVA. *P* values are marked. **e,g,** Dots represent data from a single mouse (n = 7–10 per group) and bars represent the median. Experiments were repeated at least twice.

Supplementary data fig. 6: Single-cell RNA sequencing of B cell compartments.

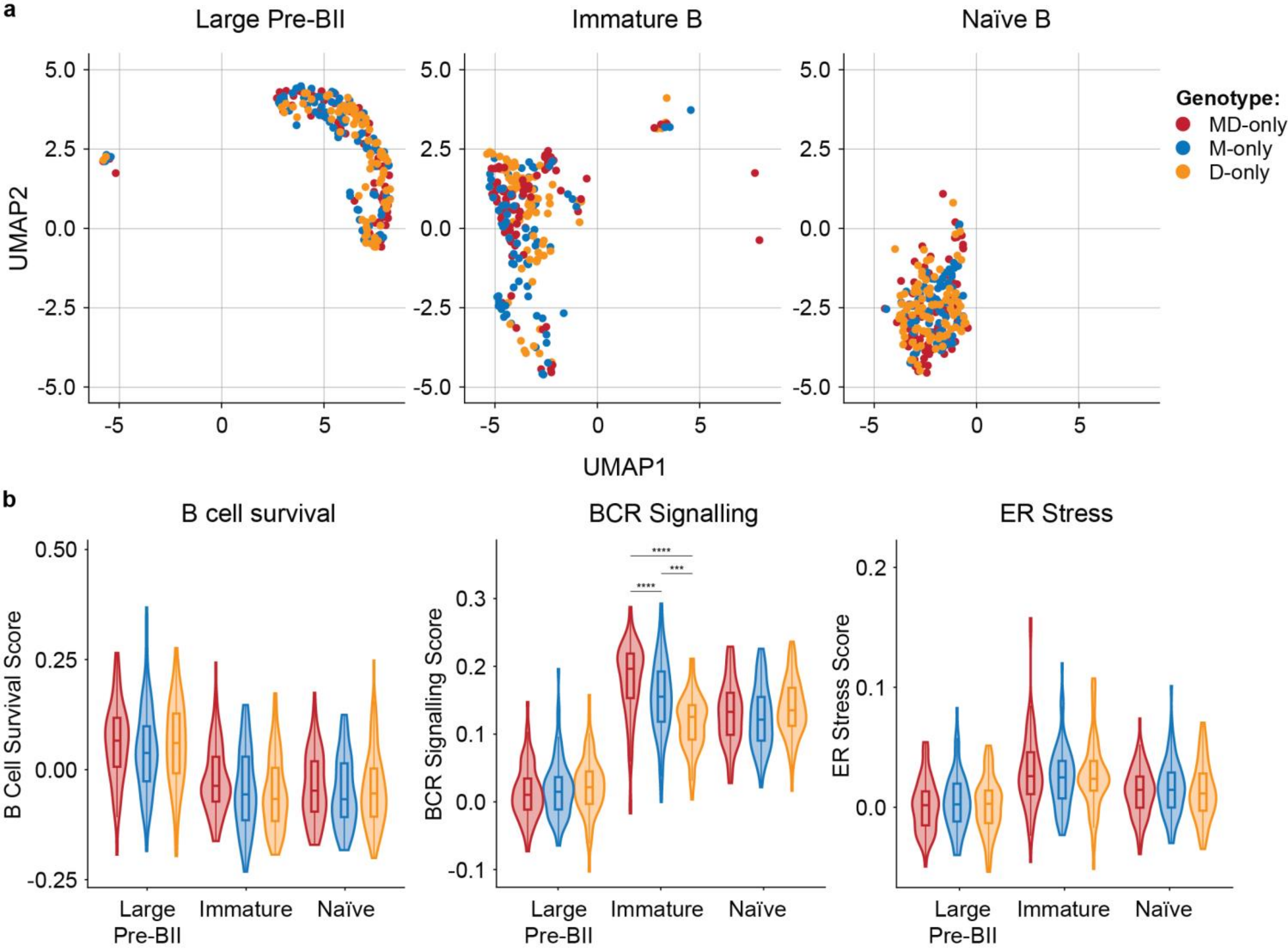

Single-cell RNA sequencing was performed on large pre-BII, immature and mature naïve B cells isolated from MD-, M- and D-only mice. **a,** UMAP plots depicting large pre-BII, immature and naïve B cell clusters. Dots represent data from a single cell, and the color denotes the genotype and B cell stage of the corresponding cell. **b,** Violin plots show gene expression pathway scores. Central boxes denote the quartile bounds. Data were compared via Mann-Whitney tests with Benjamini-Hochberg adjustment. *P*-value denotations: '***' $P < 0.001$ and '****' $P < 0.0001$.

Supplementary data fig. 7: Naive repertiore analysis of MD-, M- and D-only mice.

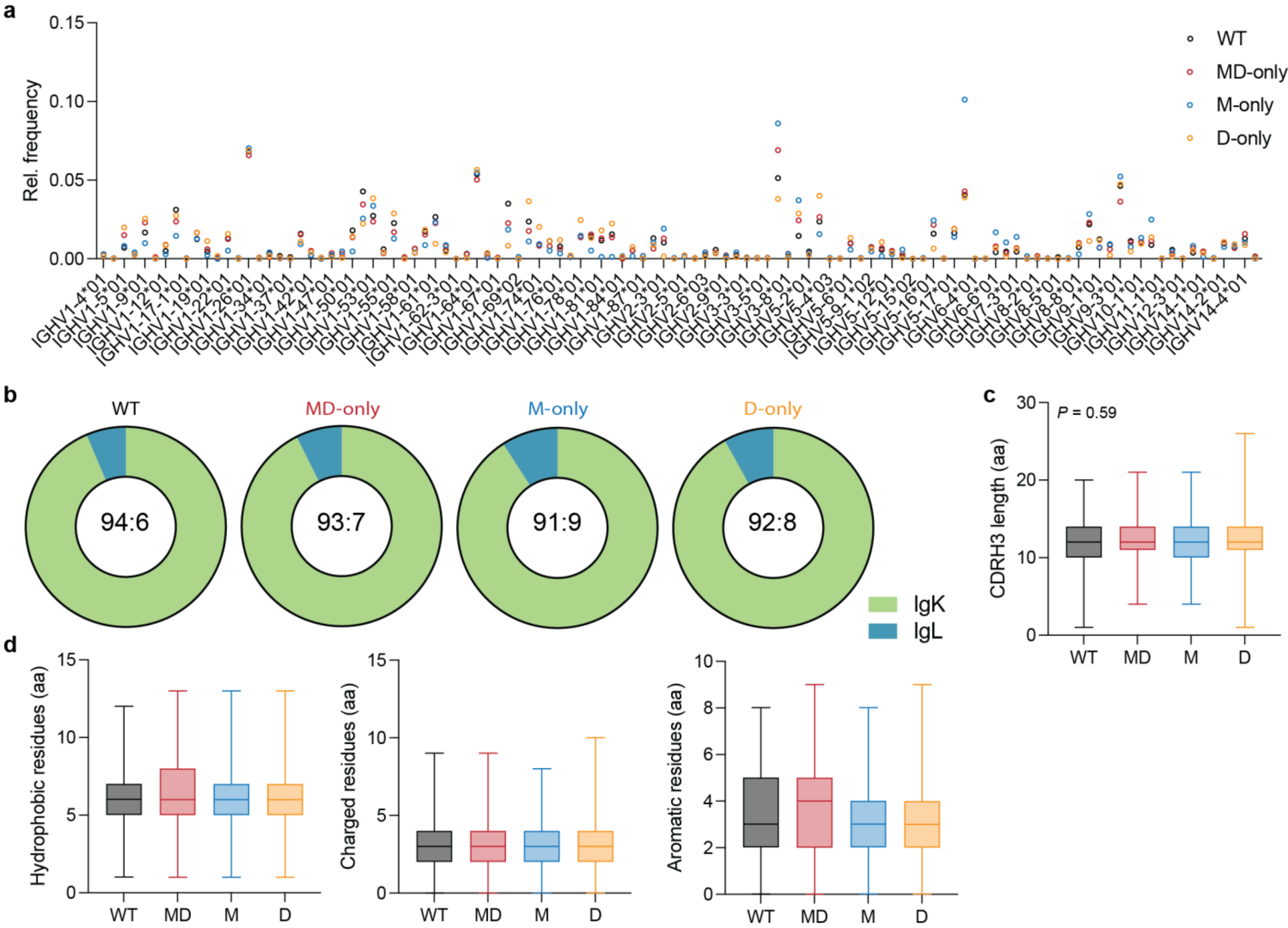

Variable regions from mature naïve (CD23^{+}CD93^{-}CD43^{-}) B cells were sequenced from MD-, M- and D-only mice (3 mice pooled per group). **a,** Plot shows the relative frequency of *Ighv* gene segments for each genotype. **b,** Pie charts show the ratio of IgK and IgL transcript expression. **c,** Box plot shows the quartiles of CDRH3 lengths of naïve B cells with respect to genotype and **d,** shows the number of hydrophobic, charged and aromatic residues. Data were compared by one-way ANOVA.

Supplementary data fig. 8: IgD expression in peripheral B cell development.

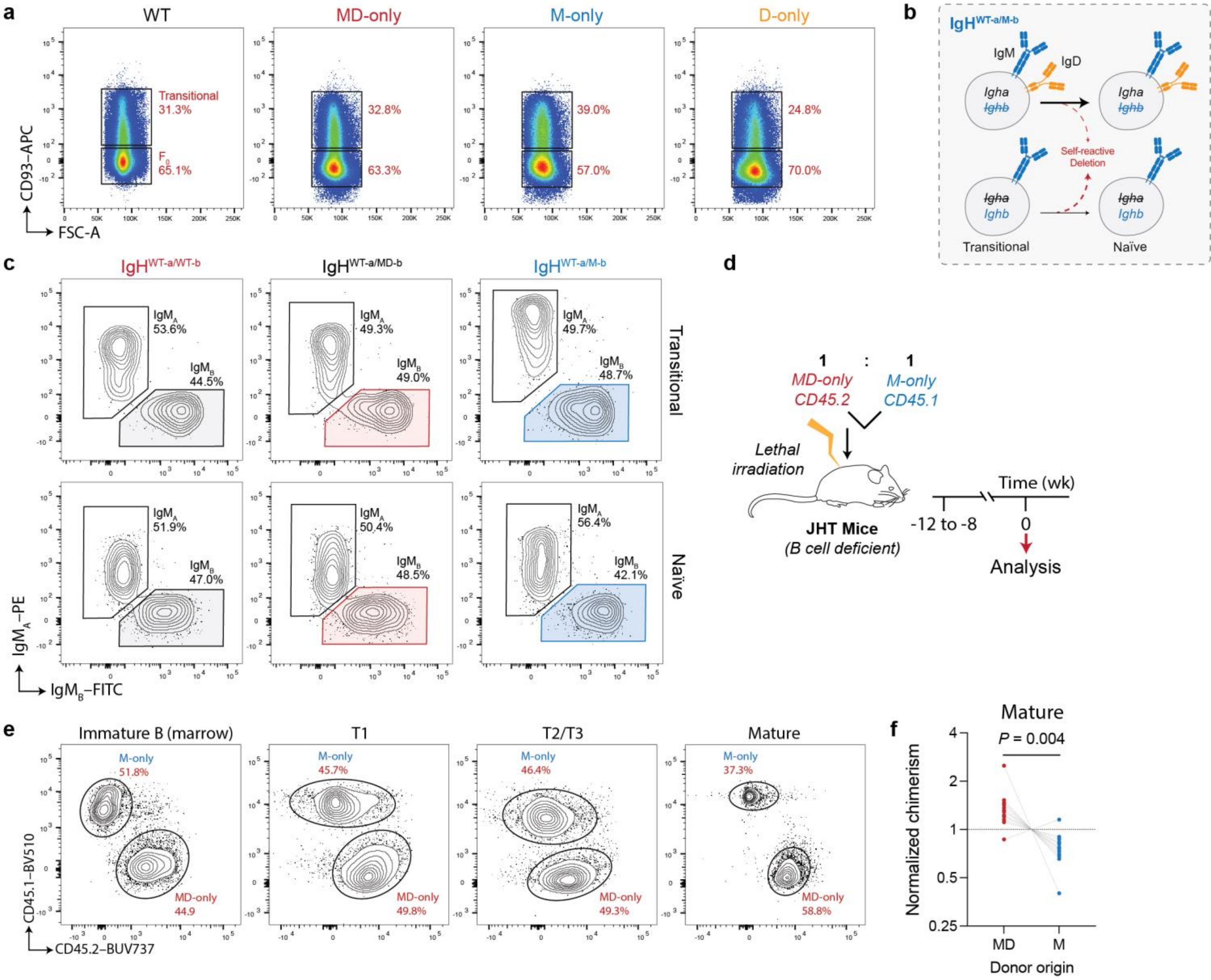

a, Representative FACS plots showing the proportion of splenic transitional and mature naïve B cells. Data were pre-gated on DUMP$^-$B220$^+$CD43$^-$ events. **b,** Diagrammatic representation of B cell peripheral deletion in IgH$^{WT\text{-}a/M\text{-}b}$ mice. **c,** Representative contour plots of allotype expression in the transitional and mature naïve B cell compartments of discordant IgH$^{WT\text{-}a/WT\text{-}b}$, IgH$^{WT\text{-}a/MD\text{-}b}$ and IgH$^{WT\text{-}a/M\text{-}b}$ mice. **d,** Diagrammatic representation of chimera production. 6–8-week-old J$_H$T mice [(48)] were lethally irradiated and bone marrow obtained from MD-only.CD45.2 and M-only.CD45.1 mixed (1:1) was administered intravenously. After 8–12 weeks, the reconstitution of B cells was evaluated by flow cytometry. **e,** Representative contour plots showing donor B cell origin, as inferred from CD45.1 or CD45.2 expression. **f,** Plots show the relative chimerism, normalized to the immature B cell compartment. Joined dots represent data from the same mouse. This experiment was repeated three independent times. Data were compared by paired t-tests.

Supplementary data fig. 9: Clone seeding and early participation of B cells in the germinal center.

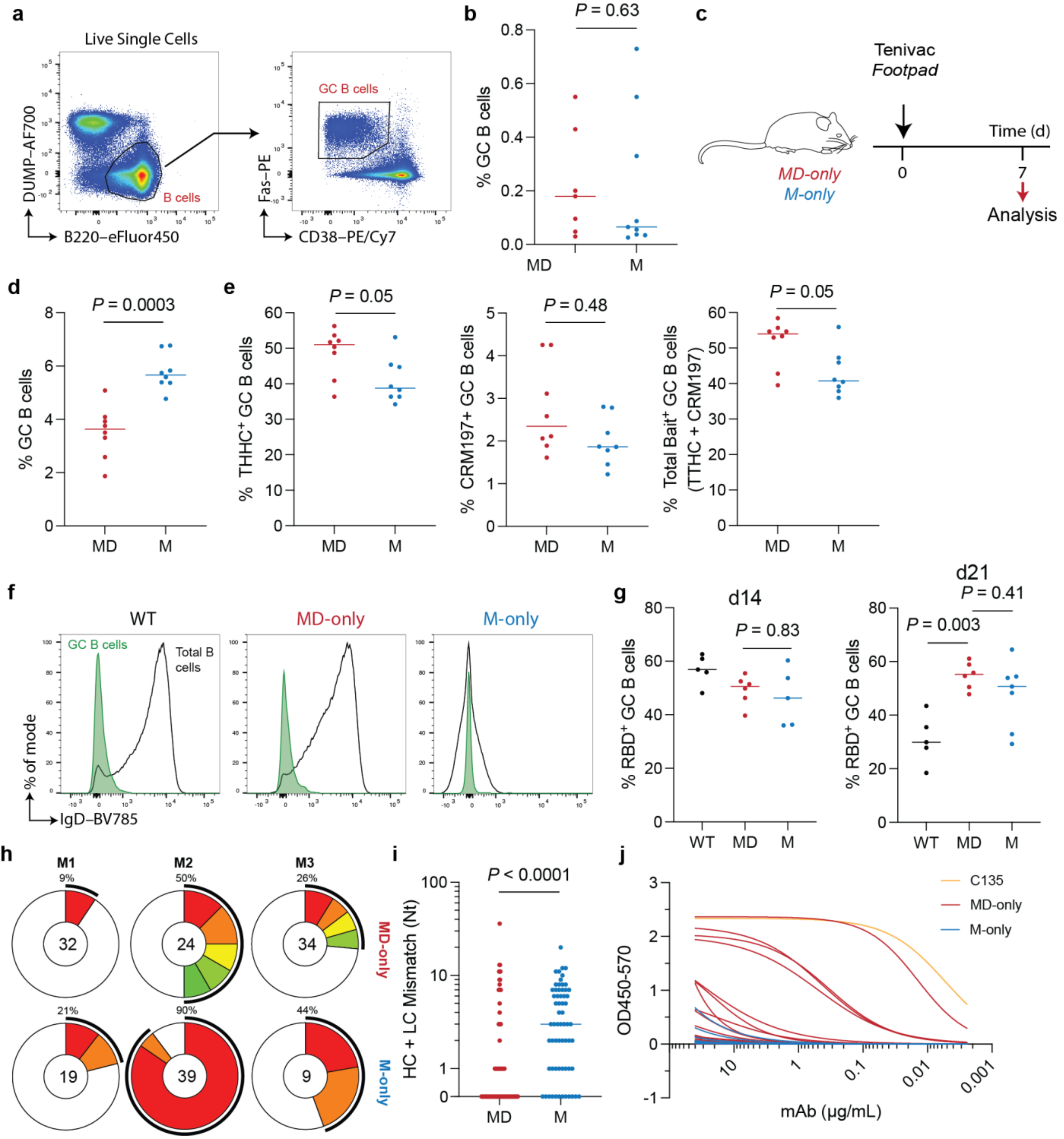

Supplementary data fig. 9: Clonal seeding and early participation of B cells in the germinal center.

a, Plots show the gating strategy for GC B cells in the pLN. **b**, Graphs show the percentage of GC B cells in mice immunized 7 days prior with NP_{17}-OVA. **c,** Immunization schedule for panels (D and E). MD- and M-only mice were immunized with Tenivac and their germinal centers were evaluated after 7 days. **d,** Plot shows the germinal center size in mice immunized with Tenivac. **e,** Plots show the percentage of Tenivac antigens THHC- and CRM197-bait-binding GC B cells post-immunization. **f,g,** Mice were immunized with RBD in alum, and fluorescently-labelled RBD bait staining of GC B cells was quantified longitudinally by flow cytometry. **f,** Representative histogram plots show the IgD expression on GC B cells 14 days post-immunization. **g,** Dot plots show the percentage of GC B cells that bound RBD molecular bait 14 and 21 days after immunization. **h,** Donut plots show the clonal distribution of paired Ig sequences (IgH and IgK/IgL) of GC B cells from MD- and M-only mice immunized 7 days prior with SARS-CoV2 RBD. Colored segments represent the proportion of cells isolated from the same inferred clonal family, whereas the white segment represents singlets. The number inside of the donut represents the number of sequences acquired from that individual. **i,** Plot shows the number of germline nucleotide mismatches in the BCR sequences of clones isolated from the GC post-immunization. Data are compared using a Mann-Whitney test. **j,** IgGs were cloned from GC B cells 7 days after RBD immunization. ELISA traces show binding to RBD. C135 Fab high-affinity positive control (yellow) was included as a positive control.

Supplementary Data Fig. 10: B cell development phenotyping of IgD^{trunc} mice.

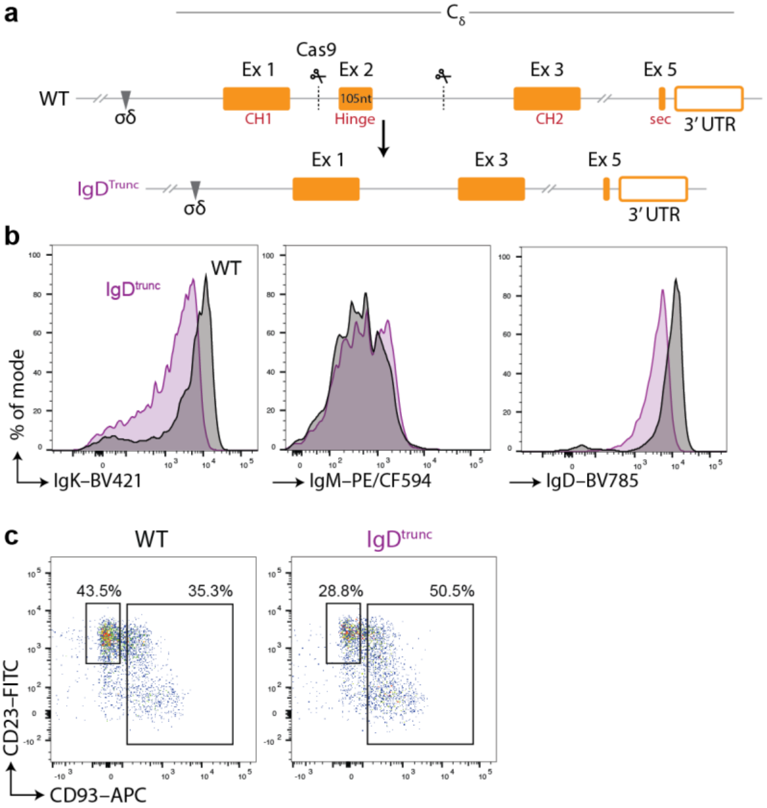

a, Graphical depiction of the C_δ region of the *Igh* loci and Cas9 gene segment excision strategy to produce the IgD^{trunc} allele by excising the exon 2, the symmetrical exon that encodes the discrete hinge portion of IgD. See methods section. **b,** Representative histograms showing the expression of IgM and IgD on B cells in the blood from both WT (black) and IgD^{trunc} mice. **c,** Representative flow cytometry plots showing the transitional and mature naïve B cell populations in mouse blood.
